# Health status and associated factors of middle-aged and older adult cancer survivors in India: results from the Longitudinal Ageing Study in India

**DOI:** 10.1186/s12885-022-10111-7

**Published:** 2022-10-22

**Authors:** Aravinda Meera Guntupalli, Y. Selvamani, Sara J. Maclennan, T. R. Dilip

**Affiliations:** 1 Institute of Applied Health Sciences (IAHS), School of Medicine, Medical Sciences and Nutrition (SMMSN) University of Aberdeen, Room 1.077, Polwarth Building, Foresterhill, Aberdeen, AB25 2ZD UK; 2 School of Public Health, SRM Institute of Science and Technology, Chennai, India; 3 International Institute for Population Sciences (IIPS), Govandi Station Road, Mumbai, 400088 India

**Keywords:** Cancer survivor, General health, Mental health, ADL and IADL limitations, Middle-aged and older adults, India

## Abstract

**Background:**

The number of persons who have survived cancer has been increasing in India as elsewhere due to advances in detection and treatment of this disease. However, evidence on the standardised number of cancer survivors, their characteristics and their complex health challenges on a national level does not exist due to data limitations. This study, therefore, examines the profile of cancer survivors and their health status using the recently released Longitudinal Ageing Study in India (LASI) survey data.

**Methods:**

LASI wave 1 is a cross-sectional nationally representative survey of 65,562 middle and older adults aged 45 and above. We first calculated the socioeconomic, demographic and geographical characteristics of cancer survivors (per 100,000 population). We later estimated the adjusted odds of poor health, sleep problems, depressive symptoms, activities of living limitations (ADL and IADL), and hospitalisation of cancer survivors using multivariable logistic regression analysis.

**Results:**

According to LASI estimates, there were 2.1 million cancer survivors in India (95% CI 1.8 million to 2.6 million) in 2017–18. Overall, 440 cancer survivors have been identified in this study, with considerable state variations. The number of cancer survivors per 1,00,000 population was relatively more in non-indigenous groups, people with a history of cancer in their families, those who worked earlier but currently not working and those in the richest quintile categories. As compared to those who never had cancer, the cancer survivors are at higher risk of hospitalisation (adjusted odds ratio (aOR) = 2.61 CI 1.86, 3.67), poor self-rated health (aOR = 3.77, CI 2.55, 5.54), depressive symptoms (aOR = 1.53, CI 1.41, 2.05) and sleep problems (aOR = 2.29, CI 1.50, 3.47). They also reported higher ADL (aOR = 1.61, CI 1.11, 2.34) and IADL (aOR = 1.49, CI 1.07, 2.07) limitations. Cancer survivors who had their cancer diagnosis in the past 2 years or a cancer-related treatment in the past 2 years have significantly higher odds of poor health status than middle-aged and older adults without a cancer history.

**Conclusion:**

Middle-aged and older cancer survivors, particularly those who underwent cancer diagnosis or treatment in the past 2 years, are at a significantly higher risk of experiencing poor self-reported health and other health challenges, suggesting the need for an integrated healthcare approach.

**Supplementary Information:**

The online version contains supplementary material available at 10.1186/s12885-022-10111-7.

## Introduction

Cancer risk has always been higher in older adults and the elderly. The projected increase in the incidence of cancer among older people (defined as aged 65 and above) indicates that this group would represent 60% of global cancer incidence by 2035 [1, 2]. Though the net survival rates for cancer patients in low and middle-income countries continue to be substantially lower than in high-income countries, there have been slow but substantial improvements in these rates, especially in the case of breast cancer between 1994 and 2004 [3]. However, the needs of the cancer survivors often get overlooked in India’s cancer control programme that is focused on cancer prevention and screening activities and in provisioning access to optimal treatment for diagnosed cases.

Cancer care services are not widely accessible across the country, and the survival of patients is also dependent on their place of residence and capacity to seek medical care. Such regional variations in cancer patients’ survival in India are well documented [4, 5]. Existing studies in India focused on the health status of cancer survivors are grounded in national health datasets that concentrate on childbearing ages. Despite the increasing percentage of middle-aged and older people living with and beyond cancer, little research has focused on understanding the physical and psychological health and wellbeing of cancer survivors aged 45 years and above.

Living with and beyond cancer places a burden both on the individuals diagnosed with cancer and the health care system. Literature from high-income countries and China suggests that cancer survivors aged 45 years and above report poor subjective health on clinically relevant aspects like self-rated health and quality of life [6, 7]. Evidence also indicates functional limitations and poor mental health, such as depression [8, 9], cognitive impairment, and dementia [10]. Cancer survivors aged 45 years and above also report higher comorbidity. This highlights a need for an integrated care pathway to avoid fragmentation of health and social care by multiple providers that handle various aspects of morbidities other than cancer [8]. To design an integrated cancer care pathway that addresses the multifaceted aspects of cancer care in India, it is crucial to improve our understanding of the physical and psychological health and wellbeing of cancer survivors.

This paper aims to study the health status of cancer survivors in India aged 45 and above and provide a comprehensive picture of the characteristics of cancer survivors in India.

## Materials and methods

### Data

The present analysis is based on the first wave of the Longitudinal Ageing Study in India (LASI) survey, a nationally representative data of 72,250 middle-aged and older adults aged 45 and above and their spouses irrespective of their age [11]. The survey was conducted by the International Institute for Population Sciences (IIPS), Mumbai, India, in collaboration with Harvard T. H. Chan School of Public Health (HSPH), USA and the University of Southern California (USC) USA. The LASI was approved by the Indian Council of Medical Research (ICMR), and informed consent, either oral or signed, was obtained from all the respondents. The LASI survey has collected data from 35 states/Union Territories (UTs) of India except for Sikkim. The LASI survey has adopted a multi-stage stratified area probability cluster sampling design within each state.

In rural and urban areas, the LASI survey has adopted a multi-stage sampling design in the selection of respondents and in the design of the sampling frame. As per the 2011 census, sub-districts were selected as a sampling frame in both rural and urban areas. Within these sub-districts, villages were selected in rural areas, and households from these selected villages were selected in the third stage. However, in each urban subdistrict, a census enumeration block is selected that acts as a sampling frame for the selection of the households. The data for the first wave used in the study was conducted in 2017–18 and released in 2021 [12]. The LASI survey has collected data on household and individual sociodemographic and economic characteristics, biomarkers and health. It is the first national-level study that collected information on work and retirement, self-reported and measured chronic health conditions, health risk factors, and functional health status for middle-aged and older adults. In the analysis, we have included the data of 65,562 participants aged 45 and above and excluded the sample of 6688 younger adults aged below 45.

### Measures

#### Self-reported Cancer

The LASI survey questionnaire had the following question with a list of chronic diseases “Has any health professional ever diagnosed you with the specific chronic conditions”. Those who reported having been diagnosed with ‘cancer or malignant tumour’ by health personnel at the time of the survey were considered cancer survivors for this analysis. The number of cancer survivors per 1,00,000 population was computed as follows to obtain the differential in the presence of cancer survivors across different subgroups of the population.

$$\frac{\mathrm{Number}\ \mathrm{of}\ \mathrm{reporting}\ \mathrm{ever}\ \mathrm{diagnosed}\ \mathrm{with}\ \mathrm{cancer}\ \mathrm{or}\ \mathrm{a}\ \mathrm{malignant}\ \mathrm{tumer}\kern0.5em \mathrm{by}\ \mathrm{a}\ \mathrm{health}\ \mathrm{professional}}{\mathrm{Total}\ \mathrm{population}\ \mathrm{surveyed}\ }X\ \mathrm{100,000}$$

#### Cancer duration and treatment period

The survey collected information on the age at which the cancer was diagnosed. Cancer duration was estimated using the respondent’s age at the time of the survey and the age of cancer diagnosis. Our duration of cancer variable had the following categories: Up to 2 years, 3–5 years and six or more years of cancer to compare and contrast health status in all these groups compared to the middle-aged and older adults without cancer. We also utilised data on cancer treatment in the 2 years preceding the survey.

#### Sociodemographic factors

Information was collected on participants’ age, sex, residence (rural or urban), educational status, and family history of cancer. Participants also provided information on caste, which was used to assess if they belonged to historically less privileged social groups such as indigenous Scheduled Tribe (ST), Scheduled Caste (SC) or the other backward class. In addition to capturing the social gradient of Indian society using the caste variable, details of their religion categorised as Hindu, Muslim, Christian or others were also noted. Households were classified as poorest, poorer, middle, richer and richest quintiles based on their monthly percapita expenditure quintile (MPCE) reported in the survey. The regional classification was carried out in the following manner to account for geographical differences: North (Chandigarh, Delhi, Haryana, Himachal Pradesh, Jammu & Kashmir, Punjab, Rajasthan and Uttarakhand), Central (Chhattisgarh, Madhya Pradesh and Uttar Pradesh), East (Bihar, Jharkhand, Odisha and West Bengal), Northeast (Arunachal Pradesh, Assam, Manipur, Meghalaya, Mizoram, Nagaland and Tripura), West (Dadra and Nagar Haveli, Daman and Diu, Goa, Gujarat and Maharashtra) and South (Andaman & Nicobar Islands, Andhra Pradesh, Karnataka, Kerala, Lakshadweep, Puducherry, Tamil Nadu and Telangana).

### Health measures of cancer survivors

#### Hospitalisation

In the LASI survey, the proportion of hospitalised was generated based on the following question “Over the last 12 months, how many times were you admitted as a patient to a hospital/long-term care facility for at least one night”? Those who said one or more times were considered hospitalised in the last year.

#### Depressive symptoms

The short 10-item scale that considers seven negative and three positive symptoms developed by the Center for Epidemiological Studies Depression **(**CESD), validated in Indian settings [13], was used to assess the prevalence of depressive symptoms. A score of 4 or more on the 10-point scale means that a respondent was experiencing depressive symptoms often or all of the time.

#### Poor self-rated health

In the LASI survey, self-rated health was assessed by the following question. “Now I want to ask you about your general health. Overall, how is your health in general? Would you say it is very good, good, fair, poor, or very poor”? In the analysis, we combined poor and very poor to represent poor self-rated health.

#### Limitations in activities of daily living (1 + ADL)

In the LASI survey, the difficulties in functional health were assessed based on problems in everyday functions, which include visiting the toilet, self-feeding, dressing, grooming, ambulation, and bathing. We combined these indicators and generated a single variable with no ADL limitations and 1+ ADL limitations.

#### Limitations in instrumental activities of daily living (1 + IADL)

The indicators included in the study to assess the instrumental activities include difficulties in preparing a hot meal, shopping for groceries, making telephone calls, taking medications, doing work around the house or garden, managing money, such as paying bills and keeping track of expenses and getting around or finding an address in an unfamiliar place. We further generated a single indicator of IADL representing no IADL limitations and 1 + IADL limitations.

#### Sleep problems

In the LASI survey, four questions were asked to assess the prevalence of sleep problems which include (1) trouble falling asleep (2) trouble getting back to sleep (3) not being able to fall asleep again (4) feel unrested during the day. Those who reported frequent experience (5 or more nights per week) for any of the four questions were considered to have sleep problems.

### Statistical analysis

Bivariate and multivariable analyses were used to fulfil the objectives of the study. Bivariate analysis was carried out to understand the share of cancer survivors across the subgroups of the population. In the absence of reliable data, the number of cancer survivors per 1,00,000 population is, to some extent, a proxy for differentials in prevalence in the respective subgroups.

Separate multivariate binary logistic regression analysis is performed to examine the factors associated with the risk of being a cancer survivor and to examine the association between being a cancer survivor and the risk of experiencing each of the six adverse health outcomes (hospitalisation, poor self-rated health, activities and instrumental activities of daily living difficulties, depressive symptoms (CESD) and sleep problems analysed in the study. Let Y be the binary dependent variable indicating the health risk coded as 1- respondent experienced the risk and 0 if otherwise and p be the probability of Y to be one, i.e. *p* = P(Y = 1). For the dependent variable, the logistic regression model takes the following general form:

$$\mathrm{Logit}\ \mathrm{P}=\ln\ \left[\mathrm{P}/\left(1\hbox{-} \mathrm{P}\right)\right]={\mathrm{b}}_0+{\mathrm{b}}_1\mathrm{x}1+{\mathrm{b}}_2\mathrm{x}2+{\mathrm{b}}_3\mathrm{x}3+{\mathrm{b}}_4\mathrm{x}4+-+{\mathrm{b}}_{\mathrm{i}}\mathrm{xi}+\mathrm{ei}$$

Where b_1_, b_2_, b_3_, −----, b_i_ represents the coefficients of each of the predictor variables × _1_, ×_ 2_ × _3_---,x_i_ included in the model and e_i_ is the error term. Ln [P/ (1-P)] represents the natural logarithms of the odds of the outcomes or risk. Results are presented as odds ratios with 95% confidence interval. We used the individual level sampling weights provided in the LASI data sets. All statistical analyses were performed using STATA 15 (Stata Corp, LP, College Station, Texas).

## Results

### Characteristics of the study population

Table 1 presents the descriptive statistics of the study population in addition to the characteristics of cancer survivors. Overall, the share of study participants is highest in the 45–54 age group, and females outnumbered males. Most of the study population resides in rural areas (69%). The majority of the study population belongs to the Hindu religion (82%), followed by Muslims. Only 6% of the middle-aged and older participants have completed college or more education. Nearly half of the study participants are currently engaged in work. More than 5% of the study population reported a family history of cancer.

**Table 1:** Sample details and cancer survivors by selected background characteristics among older adults aged 45 years and above, India, 2017–18

Background characteristics	Total sample (%)	Total number of cases (N)	Cancer survivors per 1,00,000 population (95% CI)
**Age groups**			
45–54	35.0	24,094	552 (469, 636)
55–64	29.8	20,136	657 (581, 733)
65–74	23.8	14,583	706 (517, 895)
75+	11.4	6749	735 (511, 878)
**Residence**			
Rural	68.5	42,424	543 (489, 598)
Urban	31.5	23,138	854 (710, 998)
**Sex**			
Male	45.9	30,479	484 (419, 549)
Female	54.1	35,083	773 (680, 867)
**Marital status**			
Currently married	73.3	48,769	580 (529, 632)
Others	26.7	16,793	805 (633, 978)
**Caste**			
Scheduled tribe	8.6	11,365	381 (287, 476)
Scheduled caste	19.2	10,959	572 (458, 686)
Other backward class	44.4	24,629	508 (442, 574)
Others	26.8	18,609	840 (744, 937)
**Religion**			
Hindu	82.0	48,099	624 (557, 692)
Muslim	11.5	7803	631 (502, 761)
Christian	3.0	6536	832 (547, 1117)
Others	3.5	3124	887 (620, 1155)
**Education**			
Less than primary or primary	73.7	46,123	635 (561, 706)
Middle/higher secondary	20.7	14,693	626 (523, 719)
College or above	5.6	3664	810 (581, 1033)
**Work status**			
Currently working	46.2	30,177	443 (381, 505)
Worked in the past but currently not working	27.8	17,393	825 (723, 927)
Never worked	26.0	17,992	795 (627, 963)
**Expenditure quintile**			
Lowest	20.9	12,941	496 (291, 502)
Second	21.2	13,190	468 (383, 553)
Middle	20.5	13,163	456 (386, 527)
Fourth	19.4	13,210	926 (686, 1167)
Highest	18.0	13,058	1027 (885, 1169)
**Family history of cancer**			
No	94.8	61,695	581 (521, 640)
Yes	5.2	3358	1842 (1442, 2222)
**All India**	100	65,562	641 (582, 700)

CI is the confidence interval

### Sociodemographic characteristics of cancer survivors

In India, there were 641 cancer survivors per 1,00,000 population aged 45 years above (Table 1). There are significant differences in survivors per 1,00,000 population across age groups, sex of the older adult, place of residence, and marital status. Social group variations show that cancer survivors are significantly higher in castes that never faced historical discrimination compared to other castes or indigenous tribal communities who tend to have poor access to health systems. Similarly, more cancer survivors are in the higher expenditure quintiles than in the lowest and lower quintiles.

**Fig. 1:**
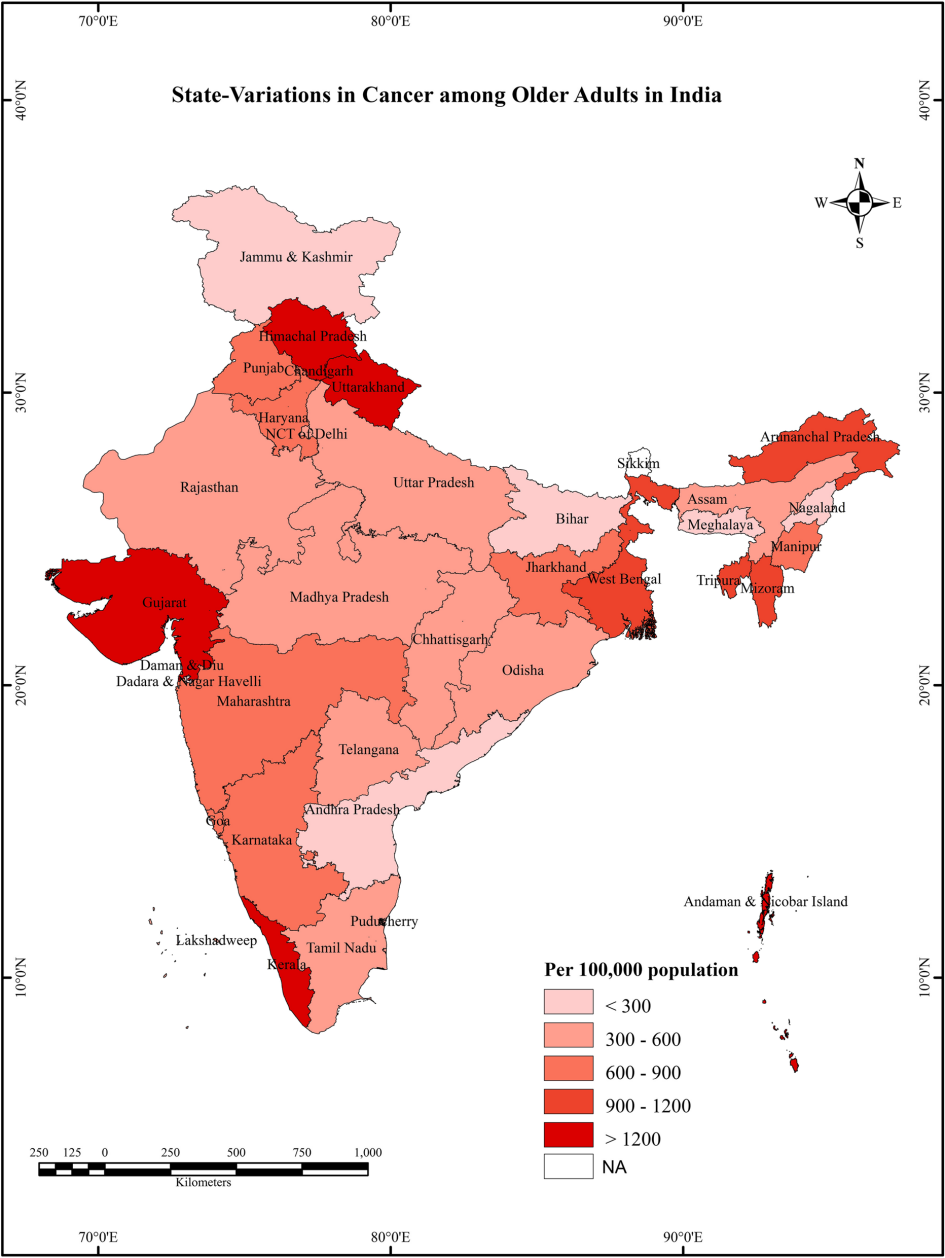
State variations in cancer among older adults in India

Survivor data clearly indicates that the survivor-to-population ratio to higher among groups with a family history of cancer (1842 per 1,00,000) than those without such a history (581 per 1,00,000). Variations in the distribution of cancer survivors indicate a substantial heterogeneity across states/UTs of India Fig. 1**.** The density of cancer survivors in the population is higher in Himachal Pradesh, followed by Kerala, which highlights better treatment and care than other states. The lowest was observed in Nagaland, a North-East Indian state.

In this study, the most common types of cancer are endometrium or uterus, followed by breast and stomach cancers (Supplementary Fig. 1). Further, 23% of cancer survivors have not received cancer treatment in the past 2 years (Supplementary Fig. 2).

The health status variables used in the study are listed (Supplementary Table 1) to provide a snapshot of the health status variables. 10% of the sample was hospitalised in the last 12 months. 28% have depressive symptoms, 18% have poor self-reported health, and 13% have sleep problems. 17 to 37% of the survey respondents reported ADL or IADL limitations.

The mean and median age at cancer diagnosis of cancer survivors shows heterogeneity in cancer care (Supplementary Table 2). Central, South and East India have a lower mean and median age of cancer diagnosis compared to India and the other regions. Our analysis does not mirror the latest literature suggesting that the median age of cancer diagnosis is lower in Northeast Indian states.

The adjusted logistic regression results of cancer survivors are presented in Table 2**.** The economic gradient of cancer survivors continued to be significant. Cancer survivors are likely to have worked in the past and are currently not working. The cancer survivors have a higher odds of family history of cancer. The study population with a family history of cancer are three times more likely to be in the cancer survivors’ group. Cancer survivors are significantly likely to be from the highest expenditure quintile and Western India.

**Table 2:** Logistic regression results of odds of being a cancer survivor among middle-aged and older adults (45+) in India, LASI Wave 1, 2017–18

**Age groups**	Adjusted Odds Ratio (95% CI)
45–54	1
55–64	1.12 (0.75 1.67)
65–74	0.83 (0.51 1.34)
75+	1.13 (0.67 1.90)
**Residence**	
Rural	1
Urban	1.29 (0.87 1.92)
**Sex**	
Male	1
Female	1.41 (0.96 2.07)
**Marital status**	
Currently married	1
Others	0.96 (0.66 1.40)
**Caste**	
Scheduled tribe	1
Scheduled caste	1.54 (0.76 3.12)
Other backward class	1.34 (0.74 2.45)
None of them	1.69 (0.91 3.11)
**Religion**	
Hindu	1
Muslim	0.99 (0.62 1.57)
Christian	1.62 (0.89 2.93)
Others	1.23 (0.62 2.43)
**Education**	
Less than primary or primary	1
Middle/higher secondary	0.80 (0.54 1.19)
College or above	0.94 (0.46 1.92)
**Work status**	
Never worked	1
Worked in the past but currently not working	1.70***(1.21 2.38)
Currently working	1.09 (0.70 1.72)
**Expenditure quintile**	
Lowest	1
Second	1.06 (0.56 1.99)
Middle	1.11 (0.60 2.05)
Fourth	1.81 (0.96 3.42)
Highest	2.67**(1.48 4.81)
**Family history of cancer**	
No	1
Yes	3.30***(2.21 4.93)
**Region**	
North	1
Central	0.75(0.45, 1.25)
East	1.41 (0.91, 2.17)
Northeast	0.88 (0.50, 1.55)
West	1.65**(1.03, 2.64)
South	0.67 (0.41, 1.10)

CI confidence interval

*** *p* < .001, ** *p* < .01, * *p* < .05

### Health and wellbeing of cancer survivors

Across all six outcome indicators, poor self-rated health, 1 + ADL limitations, 1+ IADL limitations, sleep problems and hospitalisation, the prevalence of health challenges in the last year is significantly higher among cancer survivors (Supplementary Table 3).

Table 3 presents the adjusted odds ratio of poor health-related outcomes among middle-aged and older cancer survivors compared to middle-aged and older adults without cancer. Overall, cancer survivors had poor self-rated health, depressive symptoms, functional limitations, sleep problems, and higher hospitalisation. The odds ratio of hospitalisation is two times higher among cancer survivors (adjusted OR = 2.61 CI = 1.86, 3.67, *p* < .001). The cancer survivors are more than three times more likely to report poor self-rated health (adjusted OR = 3.77 CI = 2.55, 5.54, *p* < .001) than those without a history of cancer. Similarly, the odds ratio for cancer survivors was 1.61 times higher for 1 + ADL limitations (CI =1.11, 2.34, *p* < .05), 1.49 times higher for 1 + IADL limitations (CI =1.07, 2.07, *p* < .05), 1.53 times higher for depressive symptoms (CI =1.41, 2.05, *p* < .01) than those without a history of cancer. The cancer survivors in India reported higher odds of sleep problems (adjusted OR = 2.29 CI =1.50, 3.47, *p* < .001) than those without a history of cancer.

**Table 3:** Adjusted odds ratio between health-related outcomes among middle-aged and older adults (45+) and cancer in India LASI Wave 1, 2017–18

Health related outcome	Adjusted Odds Ratio(95% CI)
Hospitalisation	2.61*** (1.86, 3.67)
Depressive symptoms	1.53** (1.41, 2.05)
Poor self-rated health	3.77*** (2.55, 5.54)
1 + ADL	1.61* (1.11, 2.34)
1 + IADL	1.49* (1.07, 2.07)
Sleep problems	2.29*** (1.50, 3.47)

*ADL* Activities of daily living, *IADL* Instrumental activities of daily living

Results are adjusted for age, marital status, place of residence, caste, religion, education, work status and MPCE quintile and region. Dependent variable coded as “1” if respondent had negative outcomes of health and “0” otherwise

CI confidence interval

*** *p* < .001, ** *p* < .01, * *p* < .05

Table 4 presents the adjusted odds ratio of poor health-related outcomes by the duration of cancer and cancer status. Overall, the odds of sleep problems and poor self-rated health were higher among all cancer survivors, irrespective of the diagnosis period. However, the odds were the highest among those diagnosed with cancer in the past 2 years preceding the survey compared to those without cancer. Cancer survivors who had a cancer diagnosis in the last 2 years preceding the survey also had a significantly higher risk of depressive symptoms (adjusted OR = 2.11 CI = 1.14, 3.90, *p* < .01), functional limitations with activities of daily living (adjusted OR = 2.16 CI = 1.24, 3.76, *p* < .01), sleep problems (adjusted OR = 3.20 CI = 1.42, 7.21, *p* < .01), and hospitalisation (adjusted OR = 5.22 CI = 3.06, 8.93, *p* < .001) compared to those who never had cancer. There is no significantly increased risk of having depressive symptoms, functional limitations with activities of daily living, and hospitalisation among those diagnosed in the last 3–5 years or 6 years and more compared to those without cancer.

**Table 4:** Adjusted odds ratio of various health-related outcomes among middle-aged and older Indian adults (45+) by their cancer duration in LASI Wave 1, 2017–18

	Adjusted Odds Ratio(95% CI)			
	No cancer (ref)	Up to 2 years of cancer	3–5 years of cancer	6+ years of cancer
Hospitalisation	1	5.22***(3.06,8.93)	1.14 (0.42,3.10)	1.52 (0.78, 2.97)
Depressive symptoms	1	2.11**(1.14,3.90)	1.86 (0.97, 3.56)	0.87 (0.54, 1.41)
Poor self-rated health	1	6.84***(3.27,14.37)	2.72*(1.21,6.09)	3.05***(1.90,4.91)
1 + ADL	1	2.16**(1.24,3.76)	1.49 (0.56,3.94)	1.51 (0.87,2.60
1 + IADL	1	1.53 (0.84,2.78)	1.72 (0.84,3.50)	1.30 (0.80,2.12)
Sleep problems	1	3.20** (1.42,7.21)	2.12*(1.03,4.35)	1.85*(1.06,3.21)

*ADL* Activities of daily living, *IADL* Instrumental activities of daily living

Results are adjusted for age, marital status, place of residence, caste, religion, education, work status and MPCE quintile and region. Dependent variable coded as “1” if respondent had negative outcomes of health and “0” otherwise

CI confidence interval

*** *p* < .001, ** *p* < .01, * *p* < .05

Table 5 looks at the association between the treatment period and health status, and the results almost mirror the results shown in Table 4. Cancer survivors who received treatment in the past 2 years are more likely to have poor self-reported health across all the measures than those without cancer. There was no significant difference between people without cancer and the cancer survivors who were not treated for cancer in the past 2 years in terms of hospitalisation, depressive symptoms, functional limitations with activities of daily living, and sleep problems. However, poor self-rated health was significantly poorer among the cancer survivors without treatment in the past 2 years compared to those without cancer (adjusted OR = 3.40 CI = 1.16, 9.95, *p* < .05).

**Table 5:** Adjusted odds ratio of various health-related outcomes among middle-aged and older Indian adults (45+) by their cancer duration in LASI Wave 1, 2017–18

	No cancer (ref)	No treatment in the past two years	Treated in the past two years
Hospitalisation	1	1.32 (0.54, 3.21)	3.08 *** (2.13, 4.45)
Depressive symptoms	1	1.44 (0.72, 2.87)	1.56** (1.12, 2.15)
Poor self-rated health	1	3.40 * (1.16,9.95)	3.92 *** (2.70, 5.70)
1 + ADL	1	1.69 (0.79, 3.65)	1.60* (1.07, 2.39)
1 + IADL	1	1.02 (0.54, 1.92)	1.68** (1.16, 2.42)
Sleep problems	1	2.92 (1.00, 8.52)	2.09*** (1.40,3.10)

*ADL* Activities of daily living, *IADL* Instrumental activities of daily living

CI confidence interval

*** *p* < .001, ** *p* < .01, * *p* < .05

## Discussion

To the best of our knowledge, this is the first study to focus on the health status of middle-aged and older adults living with and beyond cancer in India using a nationally representative survey. According to LASI estimates, there were 2.1 million cancer survivors in India in the middle and old age group (95% CI 1.8 million to 2.6 million) in 2017–18. These numbers are increasing every year due to increases in the prevalence of cancer as well as survivorship after treatment. This detailed understanding of the impact of cancer on middle-aged and older adults is essential to inform and strengthen integrated cancer care in India. Health issues of older people in India are often ignored and under-researched [14, 15]. This investigation of the health status of middle-aged and older adults living with and beyond cancer stresses the need to have better data and longitudinal datasets on cancer survivors in India to evaluate health and wellbeing, particularly among the neglected and vulnerable older adults. While the subsequent datasets of the LASI will fill this gap to some extent, there is a need to have nationally representative cohort and longitudinal databases with a specific focus on cancer. This, in turn, will support a better understanding of the critical points of unmet needs within the cancer care pathway and provision of care. It will also inform policy and commissioning of services.

The results also inform education and training programmes for the oncology workforce. Filling this gap in education and training can contribute to improvements in knowledge and skills for the provision of comprehensive care to older patients. These inequalities in workforce skills and knowledge are still a considerable challenge for low and middle-income countries (LMICs) [16].

Our study is also the first to show regional and state-level variation in cancer survivors, reflecting the heterogeneity in exposure to risk factors, behaviour and access to cancer screening at the early stages of cancer. Gender difference in self-reported cancer survivorship is notable with a high proportion of female survivors, even though females are at a higher risk of cancer due to the additional risk of breast and gynaecological cancers. This may reflect a bias in the data or may be driven by more timely healthcare engagement from women.

Cancer survival in our study is significantly higher among those in the richest expenditure quintile compared to the poorest showing the economic gradient. The positive association we found between economic status and cancer is consistent with previous studies conducted among the adult population [17]. Previous studies indicate that better treatment among the population with higher socioeconomic status results in higher survival than those with lower socioeconomic status [17]. The results are also consistent with evidence collected from other high-income countries, including the US, which reported higher mortality in low socioeconomic groups [18]. Studies also highlight the association between individual and area-level/neighbourhood socioeconomic status with cancer mortality and survival [19, 20].

It is important to note that cancers associated with lifestyle factors tend to be in higher socioeconomic groups and urban areas. However, people from high socioeconomic groups and those living in urban areas have access to better health care, including cancer screening and treatment, which results in higher survival rates. We expect that this scenario will not continue forever due to changing behavioural risk factors in lower socioeconomic groups, in addition to gradual improvements in access to cancer screening and treatment.

Our results on geographical variation do not mirror the literature. For instance, Northeast India did not show considerably poorer survivors [21]. This could be due to the occurrence of aggressive forms of cancer (e.g., breast and colorectal cancer) at younger ages in this region, combined with poor access to health facilities that can screen or treat cancer [22, 23]. Western India has significantly higher odds of survivors. This could be due to better cancer care provided in Mumbai, led by Tata Memorial Hospital.

Family history of cancer is positively associated with cancer survivorship among older adults in India. This is similar to previous studies [24]. The results suggest that people with known history might carry out early screening and care compared to those without a family history of cancer, contributing to higher survivor odds. In addition, several hereditary conditions and health risk factors at the family level are also associated with cancer [25, 26]. In particular, the family history of breast cancer is higher than in other cancer [27]. From this perspective, cancer screening must consider the family history of cancer, the type of cancer and the associated risk factors at the family level.

In this study, middle-aged and older adult cancer survivors reported poor physical, functional, mental health and sleep problems. Cancer survivors who had either a cancer diagnosis or treatment in the past 2 years preceding the survey had higher odds of reporting poor health status across all the indicators. Our findings are similar to studies carried out in other high-income studies. For instance, a study based on the English Longitudinal Study of Ageing (ELSA) found that older adults diagnosed with cancer in England reported a higher likelihood of poor self-rated health, low life satisfaction, depressive symptoms, and functional limitations [6, 7]. The results are also similar to a study conducted in China which found higher functional limitations and chronic diseases among cancer survivors [8]. Few systematic reviews among the adult population have observed a higher level of depression, anxiety and suicidal symptoms among cancer survivors [28, 29]. Our findings on sleep problems are similar to other studies, including a study published on cancer survivors in the US in 2019 [30]. These results hold implications for healthcare systems in India. As cancer incidence is increasing, it is essential to understand the medium and long-term effects, including the psychological needs and to provide appropriate health interventions to improve the health and wellbeing of those living with and beyond cancer [31].

Our results also highlight the need for an enhanced integrated cancer care pathway in India to avoid fragmented health and social care provision by multiple health providers, particularly after the diagnosis or treatment. Often cancer treatment in India centres around the curative aspects of cancer care and does not tend to consider the health condition of the survivors [32]. To design an integrated cancer care pathway [33] (which typically involves case-managed multidisciplinary team care, organised provider networks and financial networks), health care professionals in India need to consider the neglected aspects of care, including the psychological wellbeing of those living with and beyond cancer, their sleep challenges and their functional ability to carry out activities independently inside and outside their home. This resonates with the WHO’s recommendation of the Integrated care for older people (ICOPE) approach and would not only increase the life expectancy of the cancer survivors but also improve their life satisfaction and quality of life [34].

There are some limitations to the study. The data used in the study are cross-sectional. Hence, we cannot study if the cancer survivors had a cancer relapse. Also, we cannot control selection bias that could be due to the aggressive nature of certain cancers. Further, the main aim of the LASI survey was to collect data on the overall health and wellbeing of the older population rather than focusing on people with cancer. Hence, the sample consists of a smaller proportion of cancer survivors, which limits disaggregated analysis by type of cancer. It is also notable that the participation of cancer survivors in the survey will be much lower than their counterparts. As the paper looked into broader survivorship that can range from 0 to 20 years rather than a standardised 1- or 5-year survival -cancer, we assume this will bias the analysis of self-reported health. To address the heterogeneity among cancer survivors, we have looked into survivors who had cancer diagnoses or treatments in the past 2 years in the latter part of the analysis.

India has a relatively lower risk of cancer prevalence and has poor cancer survival rates due to limited access to cancer care, including late detection of cancer in patients. Hence one may not expect a large number of cancer survivors in a large nationally representative survey like the LASI survey. However, the authors consider this the only nationally representative population-level data set available to study the health issues among cancer survivors in older adults in India. The fact that the study results indicate a clear pattern and consistency of characteristics of a cancer survivor and the adverse health outcomes in cancer survivors strengthens this claim. Self-reported data could create bias in that those with more health awareness are likely to report the health outcomes compared to those with poor awareness. Our analysis stresses the need to have more research on cancer survivors, including relapse, particularly among the older population, to effectively understand the general health and wellbeing of the growing older population in India.

## Conclusion

To conclude, this study draws attention to the poor physical and psychological health status of middle-aged and older adult Indian cancer survivors, particularly those diagnosed with cancer or treated for cancer in the past 2 years. This is of particular importance given the projected increase in numbers over the next 10 years and the need for better integrated cancer care to manage this burden. Public policy interventions to improve the wellbeing of the growing older population should monitor the overall wellbeing of the older adults who completed treatment for cancer. This also highlights the need for better data and for an integrated cancer care pathway in India.

## Supplementary Information

**Additional file 1: Supplementary Fig. 1.** Percentage distribution of type of cancer reported by the cancer survivors aged 45 years and above in India, 2017–18. **Supplementary Fig. 2.** Percentage distribution of cancer survivors who have been treated for cancer in the last two years from the date of the survey. **Supplementary Table 1.** Descriptive statistics of the health status variables. **Supplementary Table 2.** The mean and median age of diagnosis of cancer by the region of residence. **Supplementary Table 3.** Health status, life satisfaction and hospitalisation of cancer survivors aged 45 and above in India, LASI Wave 1, 2017–18.

## Data Availability

The datasets analysed in the current study are available in the LASI IIPS repository, [LASI - Data | International Institute for Population Sciences (IIPS) (iipsindia.ac.in)] [12].
