# Supplementary material for: Health status and associated factors of middle-aged and older adult cancer survivors in India: results from the Longitudinal Ageing Study in India
Source: BMC Cancer. 2022 Oct 22;22:1087. doi: 10.1186/s12885-022-10111-7 (PMC9587652; doi:10.1186/s12885-022-10111-7)
Supplement: Supplementary file 1 — Additional file 1: Supplementary Fig. 1. Percentage distribution of type of cancer reported by the cancer survivors aged 45 years and above in India, 2017–18. Supplementary Fig. 2. Percentage distribution of cancer survivors who have been treated for cancer in the last two years from the date of the survey. Supplementary Table 1. Descriptive statistics of the health status variables. Supplementary Table 2. The mean and median age of diagnosis of cancer by the region of residence. Supplementary Table 3. Health status, life satisfaction and hospitalisation of cancer survivors aged 45 and above in India, LASI Wave 1, 2017–18. [file 12885_2022_10111_MOESM1_ESM.docx]

**Supplementary Fig.1**

Percentage distribution of type of cancer reported by the cancer survivors aged 45 years and above in India, 2017-18

**Supplementary Figure 2**

Percentage distribution of cancer survivors who have been treated for cancer in the last two years from the date of the survey

**Supplementary Table 1: Descriptive statistics of the health status variables**

| **Health status** | (%) |
| --- | --- |
| **Hospitalisation** |  |
| No | 89.9 |
| Yes | 10.1 |
| **Depressive symptoms** |  |
| No | 71.8 |
| Yes | 28.2 |
| **Poor self-rated health** |  |
| No | 81.6 |
| Yes | 18.4 |
| **1+ ADL** |  |
| No | 83.1 |
| Yes | 16.9 |
| **1+ IADL** |  |
| No | 62.8 |
| Yes | 37.2 |
| **Sleep problems** |  |
| No | 87.1 |
| Yes | 12.9 |
| **All India sample size** | **410** |

**Supplementary Table 2: The mean and median age of diagnosis of cancer by the region of residence**

| Region | Mean age of diagnosis | Median age of diagnosis |
| --- | --- | --- |
| Central | 49 | 50 |
| South | 52 | 49 |
| East | 53 | 49 |
| Northeast | 55 | 56 |
| West | 56 | 57 |
| North | 58 | 58 |
| **India** | **54** | **52** |

**Supplementary Table 3**

Health status, life satisfaction and hospitalisation of cancer survivors aged 45 and above in India, LASI Wave 1, 2017-18

|  | Population without history of cancer (%) | Cancer Survivors (%) | P value |
| --- | --- | --- | --- |
| Hospitalisation | 10.0 | 23.2 | < .001 |
| Depressive symptoms | 28.0 | 33.2 | 0.183 |
| Poor self-rated health | 18.2 | 40.8 | < .001 |
| 1+ ADL | 16.7 | 26.5 | 0.0029 |
| 1+ IADL | 37.0 | 50.3 | 0.006 |
| Sleep problems | 12.9 | 24.8 | < .001 |
| Total cases (N) | 64,949 | 440 |  |

ADL: activities of daily living

IADL: instrumental activities of daily living
